# Semiarid ethnoagroforestry management: *Tajos* in the Sierra Gorda, Guanajuato, Mexico

**DOI:** 10.1186/s13002-017-0162-y

**Published:** 2017-06-12

**Authors:** Vincent M. Hoogesteger van Dijk, Alejandro Casas, Ana Isabel Moreno-Calles

**Affiliations:** 1 Instituto de Investigaciones en Ecosistemas y Sustentabilidad, Universidad Nacional Autónoma de México (UNAM), Antigua Carretera a Pátzcuaro 8701, Col. San José de la Huerta, 58190 Morelia, Michoacán Mexico; 2 Escuela Nacional de Estudios Superiores Unidad Morelia, Universidad Nacional Autónoma de México (UNAM), Antigua Carretera a Pátzcuaro 8701, Col. San José de la Huerta, 58190 Morelia, Michoacán Mexico

**Keywords:** Agrobiodiversity, Arid zones, Biocultural heritage, Mesoamerica, TEK

## Abstract

**Background:**

The semi-arid environments harbor nearly 40% of biodiversity, and half of indigenous cultures of Mexico. Thousands of communities settled in these areas depend on agriculture and using wild biodiversity for their subsistence. Water, soil, and biodiversity management strategies are therefore crucial for people’s life. The *tajos*, from Sierra Gorda, are important, poorly studied, biocultural systems established in narrow, arid alluvial valleys. The systems are constructed with stone-walls for capturing sediments, gradually creating fertile soils in terraces suitable for agriculture in places where it would not be possible. We analyzed biocultural, ecological, economic and technological relevance of the artificial oasis-like *tajos,* hypothesizing their high capacity for maintaining agricultural and wild biodiversity while providing resources to people.

**Methods:**

We conducted our research in three sections of the Mezquital-Xichú River, in three communities of Guanajuato, Mexico. Agroforestry management practices were documented through semi-structured and in-depth qualitative interviews. Vegetation composition of local forests and that maintained in *tajos* was sampled and compared.

**Results:**

*Tajos* harbor high agrobiodiversity, including native varieties of maize and beans, seven secondary crops, 47 native and 25 introduced perennial plant species. Perennial plants cover on average 26.8% of the total surface of plots. *Tajos* provide nearly 70% of the products required by households’ subsistence and are part of their cultural identity.

**Conclusions:**

*Tajos* are heritage of TEK and land management forms of pre-Columbian Mexican and Mediterranean agricultural techniques, adapting and integrating modern agricultural practices. *Tajos* are valuable biocultural systems adapted to local semiarid conditions and sources of technology for similar areas of the World.

## Background

Steep slopes, severe long dry seasons, low rainfall, scarce plant cover, and shallow soils are common features of landscape dry areas of Mexico, a Country with nearly 60% of its territory occurring in arid, semi-arid and sub-humid environments. These areas, however, lodge about 40% of the biodiversity of the country and most endemic species [1]. In addition, these areas, harbor high human cultural richness, being inhabited by 30 of the 58 main indigenous ethnic groups identified in the country [2]. Thousands of communities settled in arid and semi-arid areas of Mexico depend on agriculture and use of local wild biodiversity for their subsistence [3]. In such context, the construction of water control, irrigation systems and strategies for preserving both water and soils such as the agroforestry terraces that allow favorable farming conditions, have been historically main worries of peoples practicing agriculture in arid lands [4]. Although studies examining peasant agroforestry land management in semiarid lands of Mexico are scarce, some of them have documented that these systems involve management of wildlife and agro-biodiversity. In the arid zones of this country, Altieri and Toledo [5], for instance, described agroforestry terraces belonging to the *Ñahñú* people of the Mezquital Valley, Hidalgo and the Papago communities in the Sonoran Desert showing a significant amount of biodiversity management involved in the systems. Our research group conducted studies in the Tehuacán Valley, Puebla with different agroforestry systems (AFS) that associate maize production with columnar cacti, rosetophyllous scrub, chaparral, mesquite, and palm forests handled by Náhuatl, Popoloca, Cuicatec, Mixtec, Ixcatec, and Mestizo peoples of the Tehuacán Valley [6–9]. Also outstanding are the *oases* managed by people in Baja California [10, 11] and the *desert gardens* in San Luis Potosí [4], and the *huamil* system in the region of the Valley of Santiago, Guanajuato [12], which associates *Opuntia* species with maize [13]. The common finding of all these studies is that AFS of arid and semiarid zones provide ecosystem benefits such as refuges for pollinators and seed dispersers, bridges that at landscape and plot level contribute to maintain the regional biodiversity, prevent soil erosion, and maintain water and soil storage. At the same time, AFS contribute to decrease the risk of losing supply of natural resources for satisfying livelihoods. In addition, these systems contribute to form complex and heterogeneous landscapes with high agro-biodiversity, which help to reduce deforestation for establishing new agricultural areas, making easier the conservation and restoration the bio-cultural diversity [14, 15].

The *tajos*, practiced by people of the Sierra Gorda, central Mexico, are highly dynamic and important agroforestry systems that however have been scarcely studied. These AFS involve irrigation and soil management techniques using the ground, sand and organic materials carried by the river, and are specifically designed by local people, according to the surface allowed by the alluvial areas, slope inclination, and other environmental conditions. *Tajos* are constructed in the lower and arid parts of the mountainous region of the northeastern Sierra Gorda, in the area considered in the past to be the northern boundary of Mesoamerica, just in the frontier with the cultural region called Aridoamerica [16]. Agroforestry and hydraulic management of the river sediments make possible that *tajos* become land for agriculture where natural conditions do not allow it. This study analyzes the ecological, economic and cultural importance of the *tajos* in the Sierra de Xichú, Guanajuato, Mexico. We particularly explored the following questions: i) Which are the main wild and domesticated components of *tajos,* the diversity and composition of perennial plant species, ii) what is the role of *tajos* in maintaining the household economy of farmers who manage them, and iii) what is the current cultural significance and potential of these forms of AFS management. Our preliminary observations of the systems allowed hypothesizing that these systems maintain significant local biodiversity and are the basis for subsistence of local people.

## Methods

### Study area

*Tajos* are in the municipality of Xichú, Guanajuato, in the mountainous region of the Sierra Gorda, which is part of the Sierra Madre Oriental of Mexico. The physiography of this region is characterized by pronounced mountains forming deep canyons, with semi-arid intermountain valleys, and small alluvial valleys (locally called *vegas*) on the banks of streams and rivers. There are some plateaus and small areas of hillocks, which are inaccessible for obtaining natural resources. The area still harbors well-conserved natural vegetation that has remained at low impairment [17–19], reason the Mexican Government decreed the Biosphere Reserve Sierra Gorda in February 2007 [20].

**Fig. 1:**
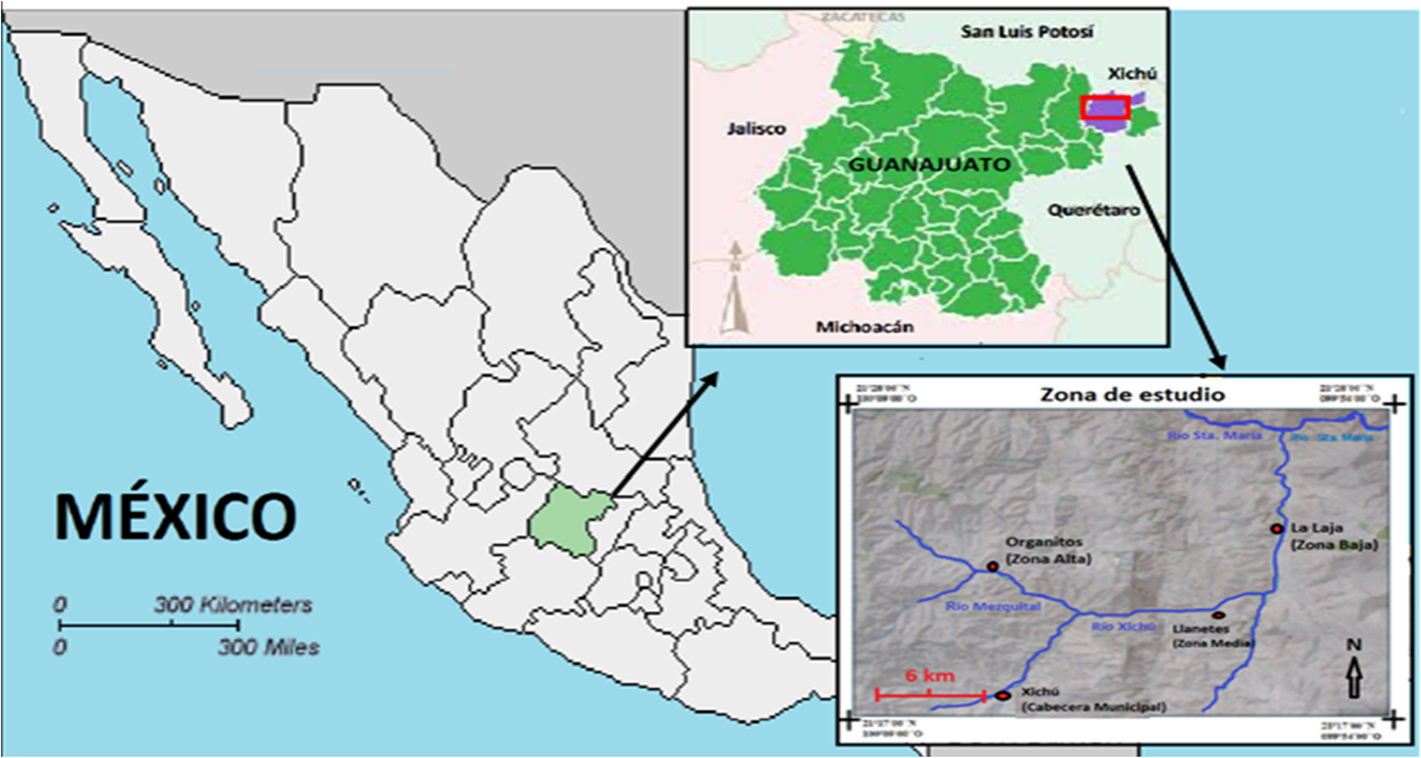
Study area. Location of the Municipality of the Xichú River, and the communities studied of Llanetes, Organitos and La Laja, in the state of Guanajuato

**Fig. 2:**
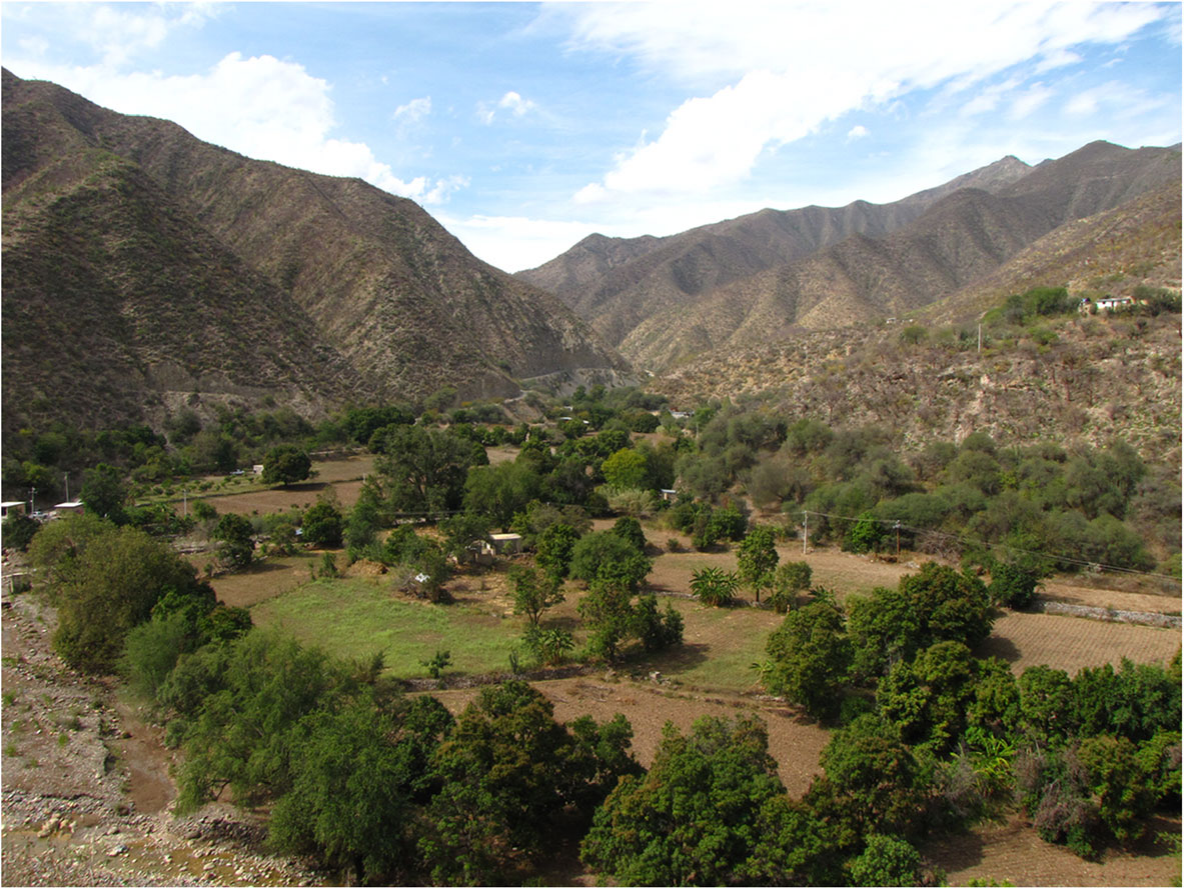
Photograph illustrating the aspect of the tajos in the study area

The study area includes the rural communities of Organitos, Llanetes, and La Laja (Fig. 1). Organitos is in the upper river area, at an elevation of 1200 m, Llanetes is in the middle part at 930 m and La Laja is in the lower area (<900 m). The communities belong to the Ejido Las Ajuntas, conformed by scattered hamlets located mainly on slopes above the floodplain (Fig. 2). The community of Organitos is composed only by 30 households, while La Laja is inhabited by nearly 100 households, according to the local authorities.

**Fig. 3:**
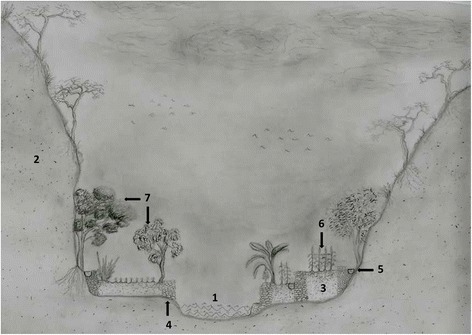
Profile of the river basin section and the location of tajos describing tajos. 1) River, 2) mountain slope, 3) *lamedal*, 4) *tajos* stone-walls, 5) *acequias*, 6) crops, 7) perennial vegetation

Climate of the study area is subtropical dry, semi-arid, influenced by the rain shadow caused by the mountains surrounding the lower parts of the basin, with average annual temperatures from 20 °C to 29 °C and annual rainfall varying from 450 to 600 mm [17, 18]. Soils are mostly shallow lithosols, except in the agricultural areas of alluvial valleys where these are formed by luvisols resulting from artificial sediment capture [19, 21]. In the study, it is possible to distinguish two environmental units: The alluvial valley formed by the passage of the Mezquital-Xichú River, and the steep slopes and ravines surrounding this valley (Fig. 3).

Local people practice the agriculture of *tajos* where the original vegetation is gallery forest surrounded by steep and barren slopes and ravines dominated by shallow soils of limestone and vegetation represented mainly by tropical deciduous forest (TDF) and rosetophyllous scrub (RS) [17, 21].

### Documenting agriculture and agroforestry practices in *tajos*

For selecting the study plots we considered that: i) the plots were in the alluvial valleys, had constant or seasonal irrigation through channels with ephemeral diversion dams deliberately constructed for agriculture; ii) the system combined crops with forest components and livestock; iii) the systems were under active management by local traditional people. We carried out in-depth qualitative interviews with farmers who manage the plots, using methods developed by Moreno-Calles et al. [7] and García-Moya [19].

### Vegetation sampling and analyses of plant diversity in the *tajos*

We described the plant diversity and structure of the *tajos*, by using the vegetation sampling method developed by Moreno-Calles et al. [7]. We sampled vegetation in nine plots, three located in each study zone. In each site, we conducted the following activities: (i) Description of the plots, geo-localization, elevation, total area and relative surface of agroforestry practices. (ii) Description of environmental aspects and agricultural management practiced in the plot. (iii) Censuses of perennial vegetation >1 m height, recording common and scientific names, and forms of use and management of each species. (iv) Measuring the diameter at breast height (DBH), height and cover of each individual plant. (v) Collecting and preparing of voucher specimens, which were deposited in the Herbarium of the Botanic Garden "El Charco del Ingenio A. C." in San Miguel de Allende, Guanajuato. The percentage of vegetation cover of the plots was calculated through the methods developed by Moreno-Calles et al. [6, 7] using polygons (rectangular, circular and triangular) to estimate the area occupied by perennial vegetation for each type of agroforestry practice (calculated by using a GPS) in each sampled plot.

To compare the plant species richness among sampled plots, we built plots using the rarefaction method developed by Gotelli and Colwell [22], using EcoSim700 [23]. The scientific names of plant species were determined according to Martinez [24] and Rzedowski et al. [17]. We standardized the plant nomenclature and authorities by using the International Plant Names Index [25].

## Results

### Tajos: concept and construction

**Fig. 4:**
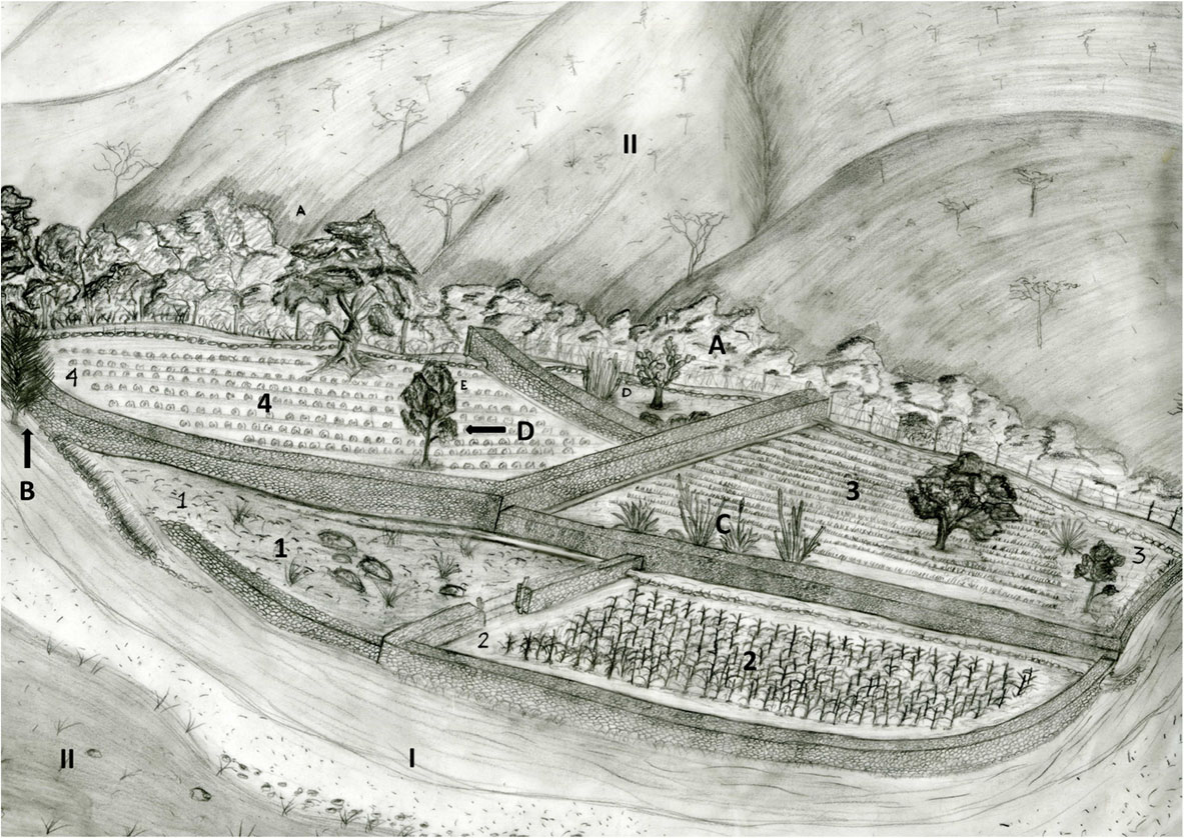
General scheme of tajos. I) River, II) mountain slope. Sequence of construction of tajos: 1) Stage 1, starting of *tajo* construction, 2) stage 2, first crops developed, 3) stage 3, older tajos with longer management, 4) stage 4, fully developed tajos with perennial plant species and crops. Perennial vegetation: A) Vegetation near slopes, B) vegetation near riverside, C) vegetation in plot boundaries, D) vegetation isle

*Tajos* are deliberate human constructed systems, involving local traditional knowledge and adaptive management of natural resources in small riversides converted into oases surrounded by semiarid landscapes. Such artifices allow practicing agriculture as agroforestry systems. In the communities studied, the term *tajo* is a polysemic term, often used as a synonym of agricultural plot or as maize-field. The word *tajo* also, makes specific reference to the stone-wall built for forming terraces near the river. Building *tajo* plots is a process based on the artificial capture of fertile river sediment (*lamedal*). The *tajos* are the only sites that have fertile soils to allow growing agricultural crops in the zone. The river valley areas where *tajos* are absent, usually are sand beaches exposed to flashfloods, narrow gallery forests or steep cliffs and slopes with very shallow and infertile soils, where it is not possible to carry out agriculture. Building *tajos* begins with the assessment of the riverbanks and mudflats adequate for its establishment. The places selected should be protected from the river flashfloods during the rainy season, but the protecting barriers should allow the capture and storage of water and sediments by gravity, gradually forming high fields that change the topography and the natural flow of water. Most *tajos* are directly next to each other, parallel to the main river stream. The plots size is variable, as well as its geographical orientation and its vegetation structure (Fig. 4). The average size of *tajos* plots was 1.2 ± 0.95 ha, but the tendency is that the plot size gradually increases down the river.

The *tajos* have infrastructural components that are common and essential for their operation. Stone-walls that surround the lower parts of the plot are the main element. These are built on the sandy river beds, using stones from the river most commonly without cement. The foundation walls are constructed with big stones to provide strength and durability to the wall. As the walls rise, people add progressively smaller stones. The main function of the walls is to retain sediments washed down form the higher mountains by the river, which are rich in organic matter, minerals, and nutrients. They also set the limits of the agricultural system with the river or the neighboring plots. The walls progressively elevate their height as the soil profile increases its depth, due to the artificial trapping of *lamedal*, a process locally called *abonar* which means fertilizing. The peasants interviewed indicated that depending on the effort invested for constructing the walls and the amount of water the river carries every rainy season, the fertile soil profile might increase from 30 to 80 cm per year. Consequently, the height of the walls is a reliable indicator of the antiquity of the active management of each plot.

The irrigation channels (locally called *acequias*) are the structures that carry water and sediments from the river to the farmlands. These are built taking advantage of the natural slope of the alluvial valley by gravity. Channels are commonly constructed by leveling out the steep slopes or digging a depression on the riverbeds, often reinforced with readily available materials such as rocks, logs, branches, and ground obtained on the site. In the steep slopes the *acequias* are often sustained by stone-walls (sometimes built by using cement) to prevent them from washing away with landslides. Generally, each plot has its own channel, but it is also common to see collective use of these structures constructed, maintained, and shared by several persons working in neighboring plots. The diverter dams, called *tomas* supply the *acequias* with streaming water from the river. These dams are built with branches, sticks and stones and covered with sand or gravel, inclined upstream allowing the diversion of water. Some are constructed with stones and cement, but generally these are temporary structures that are destroyed with flashfloods during the rainy season, and require to be rebuilt each agricultural season. To protect the *tomas* and *acequias* from flashfloods, water spoilers are often built, and these are locally called *cortinas*. The *cortinas* are constructed with various materials outside the *acequias,* inclined in favor of the stream in a way that allows that the extra water and debris can spill over back into the river. People may also use gates, weirs, and other structures to regulate the volume of water in the channels and exclude objects carried by the river current that may cause damage to other structures. For protecting crops against potential damage from domestic animals (mainly cows, goats and donkeys) the plot boundaries are usually surrounded with fences. While *tajos* depend on the river inputs (water and sediments), the river also represents the highest threat to the construction, as periods of extreme rainfall and their resulting flashfloods may cause partial or destruction of the agricultural fields. For instance, many plots were destroyed during extreme rainfall in the Summer of 2005; then, their reconstruction started again. This fact illustrates that *tajos* are spatially and temporally dynamic, as the landscape in the floodplain changes through time.

### Biocultural and agricultural relevance

The presence of crops in each time depends directly on the availability and abundance of water in the river; therefore, it is possible to register crops of the same species at very different stages at the same time, as well as in different times of the year. Peasants grow three varieties of maize (“white”, *garambullo* and “red”) and other agricultural crops like *negro enredador* beans (*Phaseolus vulgaris*)*,* chickpeas, the squashes (*Cucurbita pepo* and *C. moschata*), the gourds (*Lagenaria siceraria*), watermelon, peanuts, tomatoes (*Solanum lycopersicum* and *Physalis philadelpyca*), the chili peppers (*Capsicum annuum*), among others. Agricultural products are mainly for direct consumption of local households. When the river has water throughout the year, it is possible to produce three harvests of maize, considering that the local varieties have a ninety days’ life cycle. Harvesting maize is carried out once or twice per year, usually sowing chickpeas after harvesting maize. This cultivation pattern results in short fallow periods between agricultural cycles. It is also common to see livestock (horses, mules, goats, and cows) free-grazing inside the *tajos* during fallow periods. Maize is the main crop in the area; the peasant diet and family economy largely depend on it. The cultivation of other species and perennial vegetation management seeks primarily the use of fruits, wood, and firewood. Tilling the land (planting, weeding and fallow) is mainly by using the Egyptian plough, pulled by oxen mules or horses. For optimal development of each agricultural cycle, maize needs three irrigations, one before sowing, a second three weeks after sowing and the third one when it starts to *jilotear* (producing the female inflorescence), depending on few additional external inputs. The use of fertilizers and agrochemicals was not recorded. There is low use of chemicals to control pests or weeds, often solving problems through manual or biological control means, but some farmers use chemical insecticides and herbicides. There is a common perception among peasants that as time goes by the incidence and number of pests and invasive plant species increases, attributing it to long drought periods, extreme high temperatures and lack of frosts that naturally control the incidence of pests. Among people interviewed, there is consensus that recently there are populations of insects and invasive plants that they did not know or that did not represent damage to crops in the past.

### Ecological importance

Most of the perennial plant species occurring in the *tajos* are the result of agroforestry practices through three main mechanisms: i) Tolerance or let standing of perennial plants naturally established in the plot; ii) protection against pests, herbivorous excess of humidity, radiation or shade of perennial plants naturally occurring in the plot, and iii) deliberate introduction or propagation (by sexual or asexual means) of native and exotic species. Plant species that have utilitarian or aesthetic value within the local culture are widely tolerated and most of them are native to the ecosystem of the area. The production of fruit, the quality of wood and firewood, the quality of shadow, its contribution to feeding livestock and ornamental value are all qualities that usually influence people to decide toleration of certain plant species in agricultural areas. Several species of native perennial plants are deliberately promoted and protected for utilitarian, aesthetic or cultural values while others are removed because they are inconvenient. We registered seven induced species, nine protected species, many of them being tolerated, and at least four species that are frequently removed (Table 1).

**Table 1:** Perennial plant species management in *Tajos*

Management status	Species	Reasons	Management
Removed	*Cnidoscolus multilobus* *Rhus radicans* *Ficus cotinifolia* *Pithecellobium dulce*	Stinging and competition with crops	Removed from the surface or root. Cut with an ax or machete, removed with crowbars or burned
Tolerated	*Melia azedarach* *Acacia farnesiana*	They don ´t compete with crops or cause other damage	No specific, sometimes inns or partially removed
Protected or Encouraged	*Prosopis laevigata* *Pithecellobium dulce* *Sideroxylon palmeri* *Platanus mexicana* *Senna atomaria* *Morus sp.* *Stenocereus pruinosus* *Psidium guajava* *Carya illinoiensis*	Timber, fuelwood, shade, fodder and fruits for consumption	Protected wild and domestic animals, rising of the river, winds, drought and extreme temperatures. Pruned and fertilized. Simply transplanted occasions.
Cultivated	*Persea americana* *Mangifera indica* *Citrus* spp. *Carya illinoiensis* *Musa paradisiaca* *Carica papaya* *Prunus persica*	Fruits for family consumption and sale	Acquired through exchange or purchase. Propagated seedlings in home gardens or orchads. Transplanted to privileged sites. Fertilized, watered, pruned and grafted.

The *tajos* that we studied had on average 26.8% of perennial plants cover in relation to its total surface. Strips of vegetation are also in most *tajos*, as well as scattered trees. The cover of perennial plant species in the *tajos* depends on: i) growing and propagules production in adjacent plots and slopes; ii) cultivation in the boundaries of the plots close to the river, iii) growing of vegetation in the boundary of the plots with their neighboring plots; iv) maintaining in strips of vegetation within the plots, usually on stone walls; v) growing as isolated trees in plots (Fig. 4). Nearly 45% of the vegetation cover of plots is commonly located in the adjacencies of the plots, mainly in the slopes, 29% in the adjacencies of the plots with the river, and about 10% in the adjacencies between neighboring fields (Figs. 2, 3 and 4).

**Fig. 5:**
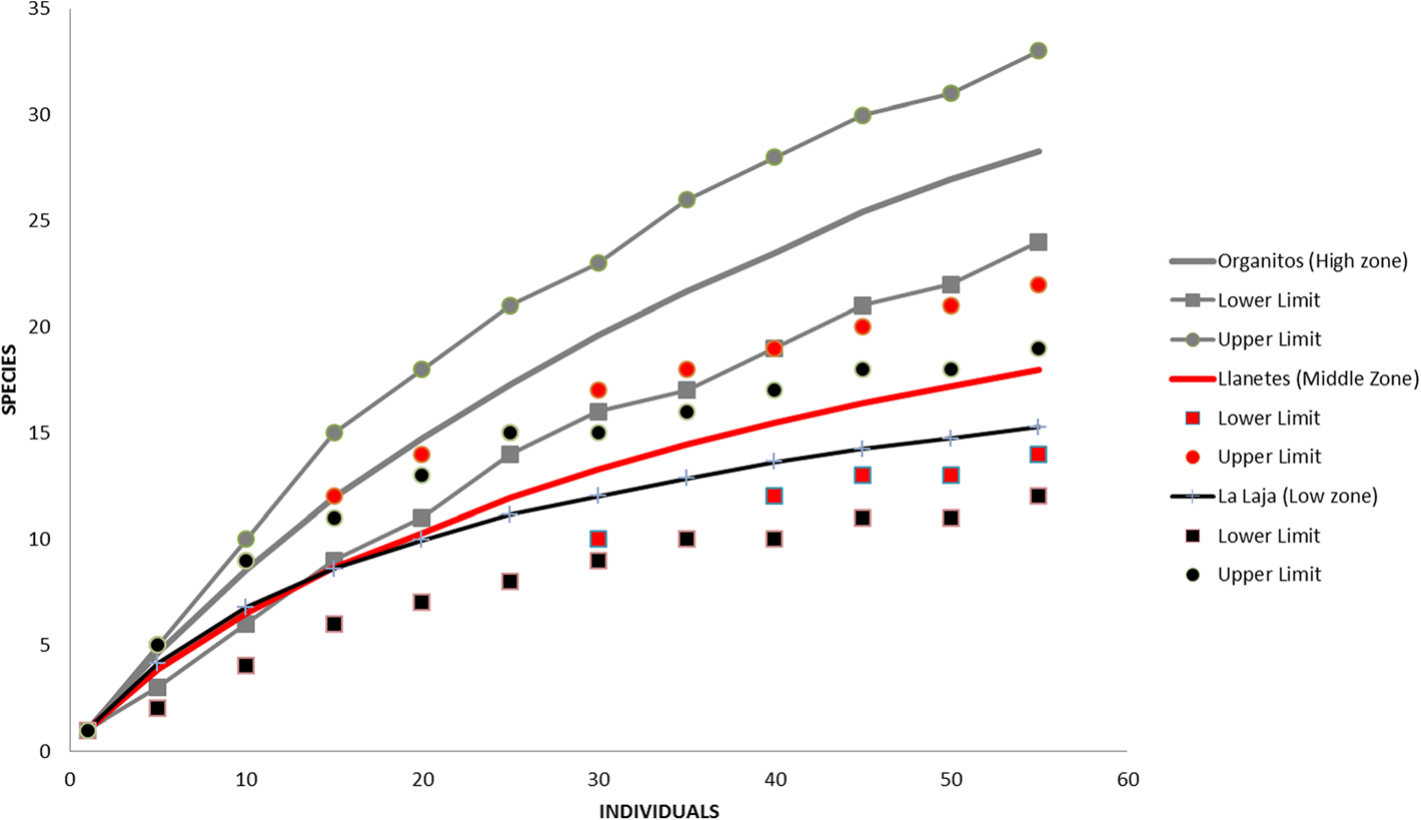
Rarefaction curves perennial plant species per community. Ratio of number of individuals and number of species expected. Differences between species richness in the *upper* area with respect to the *lower* area are observed

We recorded a total of 72 perennial plant species, 47 (65.27%) of them are native to the local ecosystems while 25 (34.72%) are exotic. In total, 49 genera of 33 different botanical families were identified (Table 2). On average, plots harbor 119 individuals of perennial plants of 18.8 species per hectare. Standardizing its surface, plots have on average 191 ± 163.77 perennial individual plants belonging to 40.22 ± 41.16 perennial plant species. The rarefaction curve analyses showed that there is a tendency that the amount of perennial plant species decreases down the river (Fig. 5). In the Organitos zone (the higher area) there are expected over 28 plant species, in the Llanetes study are (the middle area) there is an expectancy of 18 species, while in the La Laja study area (the lowest area) only 15 plant species are expected for every 55 recorded individuals (Fig. 5).

**Table 2:** Perennial species inventory

Family	Species	Local name	Origen	Life form	Uses
Agavaceae	*Agave sp.*	Maguey manso	Introduced	Agave	Sap, Fiber
Anacardiaceae	*Mangifera indica* L.	Mangoo	Introduced	Tree	Fruit
	*Rhus radicans* L.	Huau	Native	Shrub	Urticante
	*Spondias mombin* Jacq.	Ciruela amarilla	Introduced	Tree	Fruit
Asteraceae	*Montanoa sp.*	Candela	Native	Shrub	Medicinal
	*Senecio salignus* DC.	Jara amarilla	Native	Shrub	-
	*Senecio* sp.	Jarilla	Native	Shrub	-
Boraginaceae	*Cordia boissieri* A.D.C.	Trompillo	Native	Tree	Fodder
	*Heliotropium* sp		Native	Shrub	-
Budlejaceae	*Budleja* sp.	Tepozán	Native	Tree	-
	*Budleja* sp.	Tepozancillo	Native	Shrub	-
Burseraceae	*Bursera simaruba* Sarg.	Copal	Native	Tree	-
Cactaceae	*Isolatocereus dumortieri* (Scheidw.) Backeb*.*	Órgano	Native	Cacti	Fruit/Firewood
	*Opuntia imbricata* (Haw.) DC.	Cardón	Native	Cacti	-
	*Opuntia* sp.	Nopal	Introduced	Cacti	Fruit/vegetable
	*Myrtillocactus geometrizans* Console	Garambullo	Native	Cacti	Fruit
	*Stenocereus pruinosus* (Otto) Buxb.	Pitayo	Native	Cacti	Fruit
Caricaceae	*Carica papaya* L.	Papaya	Introduced		Fruit
Euphorbiaceae	*Croton* sp.	Chicharroncillo	Native	Tree	-
	*Cnidoscolus multilobus* (Pax) I.M.Johnst.	-	Native	Shrub	-
Fabaceae	*Acacia farnesiana* (L.) Willd.	Huizache	Native	Tree	Firewood
	*Acacia pennatula* Benth.	Tepame	Native	Shrub	Firewood
	*Acacia* sp.	Huizachillo	Native	Shrub	Firewood
	*Desmanthus* sp.	Mesquitillo	Native	Shrub	Firewood
	*Eysenhardtia polystachya* (Ortega) Sarg.	Palo dulce	Native	Tree	Firewood
	*Lysiloma acapulcense* Benth.	Palo arco	Native	Tree	Firewood
	*Mimosa* sp.	Garabatillo	Native	Tree	Firewood
	*Pithecellobium dulce* (Roxb.) Benth.	Huamúchil	Native	Tree	Fruit
	*Prosopis laevigata* (Humb.& Bonpl. ex Willd.) M.C.Johnst.	Mezquite	Native	Tree	Fruit, Firewod, Wood, Fodder, Ornamental
	*Prosopis* sp.	Palo gusano	Native	Tree	Ornato
	*Senna atomaria* (L.) H.S.Irwin & Barneby	Palo hediondo	Native	Tree	Firewood and shade
	*Desmodium* sp.	-	Native	Shrub	-
Juglandaceae	*Carya illinoinensis* (Wangenh.) K.Koch	Nogal	Introduced	Tree	Fruit
Labiatae	*Salvia* sp.	Salvia	Native	Shrub	Medicinal
Lauraceae	*Persea americana* Mill.	Aguacate	Introduced	Tree	Fruit
Malvaceae	*Guazuma ulmifolia* Wall.	Guazuma	Native	Tree	Firewood/shade
Meliaceae	*Melia azedarach* L.	Paradise tree	Introduced	Tree	Ornamental
Moraceae	*Ficus carica* L.	Higuera	Introduced	Tree	Fruit
	*Ficus cotinifolia* Kunth	Higuerón	Native	Tree	Ornamental/shadow
Musaceae	*Musa paradisiaca* L.	Platano jamaico	Introduced	Herb	Fruit
Myrthaceae	*Psidium guajava* L.	Guayabo	Introduced	Tree	Fruit
	-	Palo amole	Native	Tree	-
Solanaceae	*Solanum* sp.	Tomatillo	Native	Shrub	-
Platanaceae	*Platanus mexicana* Torr.	Álamo	Native	Tree	Ornamental/Fruit
Poaceae	*Arundo donax* Georgi.	Carrizo	Introduced	Herb	Handcrafts
Rhamnaceae	*Karwinskia humboldtiana*S. Watson	Sarabullo	Native	Shrub	Toxic
Rosaceae	*Prunus domestica* Thunb.	Ciruelo	Introduced	Tree	Fruit
	*Prunus persica* (L.) Batsch	Durazno	Introduced	Tree	Fruit
Rubiaceae	*Coffea arabica* L.	Café	Introduced	Shrub	Infusion
Rutaceae	*Casimiroa edulis* S.Watson	Zapote blanco	Introduced	Tree	Fruit/medicinal
	*Citrus* spp.	Lima	Introduced	Tree	Fruit
		Naranja	Introduced	Tree	Fruit
		Mandarina	Introduced	Tree	Fruit
		Limón real	Introduced	Tree	Fruit
Scrophulareaceae	-	-	Native	Shrub	-
Simaroubaceae	*Dodonaea viscosa* Mart.	Olivo	Native	Shrub	Ornamental
Ulmaceae	*Celtis pallida* Torr.	Granjeno amarillo	Native	Tree	Firewood
	*Celtis* sp.	Palo blanco	Native	Tree	Shadow
	*Celtis* sp.	Granjeno	Native	Tree	-
Verbenaceae	*Lantana camara* L.	Cinco negitos	Native	Shrub	Ornamental
Zapotaceae	*Sideroxylon palmeri* (Rose) T.D.Penn.	Capulín	Native	Tree	Fruit, wood, shade
Zygophillaceae	*Morkillia mexicana* Rose & Painter	Viuda	Native	Shrub	Ornamental

### Economic and social importance of *tajos*

About 70% of livelihoods of households interviewed depend on the products obtained from the *tajos*. Communities rely primarily of self-sufficiency agriculture (products for direct consumption of the household), and agricultural products and in smaller proportion of livestock ranching, gathering of wild species and complementary economic activities, primarily migration to urban centers and abroad in search of wage labor or local commerce business. Monetary exchange is rare and happens almost exclusively to acquire the basic products that they do not produce. While local peasants use a wide range of activities in and out of their communities, for most of them agriculture practiced in the *tajos* remains the main form of obtaining livelihoods and food security for their families throughout the year. Agricultural work is performed in groups with members of the family, by employees or as part of agreements of reciprocity, and solidarity.

Maize and beans (the most common crops) are the products mostly demanded for satisfying the basic needs of local households. Five of the nine farmers interviewed said they were self-sufficient in maize production, requiring between 1.8 to 2 tons of maize per household per year. All peasants, using on average eight to ten bags (60 kg/bag), reach self-sufficiency of beans. The demand of products depends largely on the number of members of each family, the needs of each of them and the quantity of livestock they need to feed.

Sporadic cultivation of various secondary crops such as chickpeas, squash, peanuts, tomatoes, green tomato, chili varieties, cultivation of gourds and growing fruits such as avocado and mango aims to self-consumption and small-scale sales within the community. The diversity and abundance of minor crops varies considerably between the plots sampled. Consumption of weedy plants known as *quelites* (traditional greens) contributes to the diversification and provision of edible biomass and complementary nutriments to the diet of households. Other native plant species are gathered and exploited for medicinal, food, firewood, wood for manufacturing utensils and construction, as well as for animal fodder or for aesthetic and functional values.

## Discussion

Despite the small scale of agricultural land size and the minimum proportion of municipal and regional area, *tajos* are ethonoagroforestry systems with biocultural, agricultural ecological, and socio-economic relevance like other semiarid agroforestry systems in the country and the World [5, 15].

The agricultural relevance involves maintenance of early varieties of maize (3 months) or rainfed crops resistant to drought and other crops varieties, contribute to decrease the risk of losing supply of resources for satisfying livelihoods of the Semiarid Sierra Gorda people and are alternatives of local adaptation in conditions of climatic variability and aridity. In terms of agricultural production*, tajos* having a wide variety of biodiversity in different temporal and spatial arrangements. Agroforestry practices, crop rotation, producing three crop cycles per year and temporary livestock grazing are examples of this pattern. *Tajos* artificial construction (crop terraces) for water and soil storage adapting to semiarid and variable regional and local conditions, according to the surface allowed by the alluvial areas, slope inclination, and other environmental conditions similarity in semiarid agroforestry systems in Mexico [4, 6–9, 15]. The local knowledge, practices, agricultural technology, and the associated plant species in the *tajos* are all elements of the Mesoamerican and pre-Columbian agricultural tradition, combined with many elements of the Mediterranean agricultural tradition introduced by the Spaniards. The irrigation systems accompanied by agricultural terraces allow greater control over the distribution and use of water, and is a feature shared with other pre-Columbian agricultural systems [4, 26], which is biocultural and agricultural heritage for semiarid management future.

The *Tajos* resilience is because, these AFS are highly dynamic temporally and spatially, while gradually built with stones and river sediments, they are vulnerable and recurrently destroyed by the river flashfloods during extreme rainfalls. Therefore, the alluvial valley landscape of these farming systems changes gradually throughout time. They are also adaptable, stable, and resilient over time to the bioclimatic and geomorphological conditions of the area where they are located. The adaptability to these conditions shows that the management of these AFS is the product of a long empirical tradition, which has allowed the practice of agriculture in a geographic area where natural conditions do not allow performing this activity.

*Tajos* AFS include the deliberate retention of soil, let standing, or introduction of wild trees and other woody perennial plant species to mix them with cultivated or agricultural diversity and soil and water management profitable from ecological perspectives. This pattern of around 30% of vegetation cover in agricultural plots has been seen in other semiarid regions of Mexico [6–8, 14, 15, 27]. Only nine studies in Mexico providing information about species richness in semiarid agroforestry systems, these studies have emphasized that in arid and semiarid agroforestry systems people maintain on average 69 ± 33 species of plants (SD), 71% of them native species and in regional reports are 90 (± 38) species [15]. This management provides to the systems to biodiversity at plot and landscape level [28]. Plant diversity can promote the good functionality of the system through better recycling of nutrients, greater resistance to pests and weeds, more efficient resources use, reducing the risk of resources loss, maximizing production and provision of ecosystem services like regulation of local microclimates, hydrological processes, pest control and pollination, issues that should be addressed in future studies [29]. The management of introduced and exotic plant species also contributes to increase biodiversity at the regional level, although most of the introduced elements are only occurring in the *tajos*.

Besides being the backbone of productive activities, *tajos* are key elements in the human cultural reproduction of local peasants. Within and around this agro-ecosystem, there is a large body of knowledge, practices and agricultural traditions that are a living sample of peasant culture of these communities. Around building and maintaining *tajos*, ditches and other elements of these systems, there is a huge wealth of traditional knowledge that depend on the permanence of these practices to survive and be transmitted to subsequent generations. Nature, culture and production are inseparable aspects that allow the construction of local knowledge, being the set of biotic (agro-biodiversity and associated) and abiotic (structures, tools and technologies) elements that are expressed in these AFS those who keep alive the tradition and cultural essence of the local people [30–32].

As in other regions of the warm-dry tropics, in *tajos* the key factor for growing crops is the water supply. There is a widespread notion among local people that the weather conditions have changed recently becoming extreme, causing longer drought periods and extreme warm temperatures that have complicated farming. Regional climate change, which affects water availability and increases the number of pests and invasive species, is the greatest threat to these traditional AFS and food security. The deterioration of vegetation cover and ecosystem functions and processes in the watershed and its area of influence, especially the loss and degradation of the temperate forests of the upper part of the mountains, could be the main cause of the greatest regional climate change and desertification processes in the study area than global climate change. Therefore, the future of *tajos* depends primarily on the adequate management and conservation of vegetation cover and forest areas of the mountains of the region, ensuring continuity of the ecological processes in the river basin and the provision of environmental services that result. The general loss of interest among young people in agricultural practices and the consequent aging of farmers threaten the loss of traditional farming knowledge. Promotion of information about benefits and incentives to regional agricultural production as part of public policies are therefore crucial for maintaining these ancient AFS.

## Conclusions

The *tajos* of the Sierra Gorda of Guanajuato are traditional AFS that withhold a great knowledge, techniques of management and use of local natural resources. The *tajos* are a kind of fertile artificial oases in the middle of extremely rugged and semi-arid areas where under natural conditions the practice of agriculture is impossible. With minimal use of chemical inputs, they are considerably productive, have a highly-diversified production because of hosting large agro-biodiversity and a great potential for in situ conservation of native perennial plant species, as well as local varieties of annual crops. They are the main source of economic livelihood of rural families and the backbone of the cultural identity of the communities of the region. The deterioration of ecosystems and the consequent climate change at regional and global scale, which has caused the gradual desertification and increased extreme weather events, as well as the accelerated process of acculturation, leading to the abandonment of agricultural practices and knowledge, are the main threats to *tajos* agriculture. The technological experience of *tajos* constitutes a valuable source of practices and techniques valuable for other similar arid regions of the world.
